# Evaluation of a novel virtual reality training intervention to address implicit bias among healthcare workers, using an implementation science framework

**DOI:** 10.1371/journal.pone.0331324

**Published:** 2025-10-17

**Authors:** Madelyn Olmos-Rodriguez, Lynhea M. Anicete, Nova Wilson, Luis Gutierrez-Mock, Jeremy N. Bailenson, Ali Mirzazadeh, Orlando O. Harris, Madhavi Dandu, Suzanne Welty, Alicia Fernandez, Elizabeth M. Rojo, Savanna Harris, Kelly D. Taylor, Michael J. A. Reid

**Affiliations:** 1 Institute for Global Health Sciences, University of California (UCSF), San Francisco, California, United States of America; 2 Division of Prevention Sciences, Department of Medicine, UCSF, San Francisco, California, United States of America; 3 Department of Epidemiology & Biostatistics, UCSF, San Francisco, California, United States of America; 4 Department of Communication, Stanford University, Stanford, California, United States of America; 5 Community Health Systems, School of Nursing, UCSF, San Francisco, California, United States of America; 6 Division of Hospital Medicine, Department of Medicine, UCSF, San Francisco, California, United States of America; 7 UC Global Health Institute, University of California, Oakland, California, United States of America; 8 Division of General Internal Medicine, Department of Medicine, UCSF, San Francisco, California, United States of America; JPS Health Network, UNITED STATES OF AMERICA

## Abstract

**Background:**

There is a link between racial bias and poor health outcomes among Black, Indigenous, and People of Color (BIPOC). We aimed to design and evaluate a novel pilot virtual reality (VR) training program to reduce racial bias among healthcare providers in a university healthcare system.

**Methods:**

CULTIVATE (Combatting Unequal Treatment in Healthcare Through Virtual Awareness in Empathy) is a mixed-methods study utilizing virtual reality (VR) training to enhance healthcare providers’ empathy towards racially and linguistically discordant patients. Participants included White-identifying clinical providers and graduate-level healthcare students, excluding those not engaged in direct patient care, BIPOC providers, and individuals unable to participate in VR training. Empathy was measured using a situational empathy scale and the Jefferson Empathy Scale (JSE) before and after the intervention, which comprised two VR modules, empathy assessments, and a qualitative interview. Quantitative and qualitative analyses were performed using the RE-AIM (Reach, Effectiveness, Adoption, Implementation, and Maintenance) framework to evaluate the program. The RE-AIM model will structure a framework for understanding virtual reality’s utility in future healthcare practice.

**Results:**

*Reach*: 30 adults participated, mostly women (n = 21), 47% were physicians, and 8% had no prior implicit bias training. Eighteen participants completed pre- and post-VR empathy assessments and interviews. *Effectiveness*: There was no significant difference in mean JSE scores pre- (120.7) and post-intervention (122.2), but qualitative data indicated increased emotional responses. *Adoption*: Participants endorsed the intervention with a mean score of 8 (SD = 2) on a 10-point Likert scale for recommending it to others. Implementation: The program followed the pre-designed protocol. *Maintenance*: Participants reported improved clinical empathy towards BIPOC patients in qualitative analyses.

**Conclusions:**

In this pilot study, participants found VR training to be acceptable and feasible. A larger sample needs to participate in the intervention to determine effectiveness. Qualitative results demonstrated that VR training enhanced empathy.

## Introduction

Racial bias, an unconscious negative attitude against a specific racial group, has multilayered, adverse impacts on health outcomes. Mortality rates among varying racial/ethnic groups reveal profound health disparities. Approximately two in ten Hispanic (23%) and Black (21%) adults report fair or poor health status compared to White adults (16%) in the United States [1]. Virtual reality (VR), as a technology, allows participants to explore, interact, and manipulate computer-generated real or artificial three-dimensional multimedia sensory environments in real-time, and is increasingly recognized as a powerful tool enabling healthcare professionals (HCPs) to acquire practical knowledge or skills relevant to clinical practice [2]. While extensive research has highlighted the value of VR to enhance the acquisition of technical skills [3] and procedural competencies [4,5], there is also growing recognition of the utility of VR to enhance HCP empathy since it allows individuals to viscerally experience many situations from another person’s point of view [6–10]. VR systems block out the perceptual input from the real world and replace it with perceptual input from a virtual environment that surrounds the user, is fully immersive to the user’s actions, and elicits feelings of presence. Because of these affordances, VR allows users to vividly and viscerally experience situations as if they were happening from any perspective [7]. As such, US researchers have evaluated using VR as a training tool to advance conversations regarding the consequences of discrimination on health outcomes, about racism and inequity among HCPs [6,11], and to improve empathy among medical students [12], while investigators in Hong Kong have demonstrated its value in enhancing intercultural sensitivity between persons of different race and ethnicity [12]. Given the profound and deleterious impact of systemic racism from healthcare providers on health outcomes for Black, Indigenous, and People of Color (BIPOC), there is an urgent need to evaluate innovative training interventions to increase provider empathy to this community, which is at greatest risk of harm from the racism they experienced in the healthcare system [13]. The RE-AIM framework offers an evidence-based approach for considering real-world applicability in healthcare. These insights may shape capacity building among HCPs and build on future trainings that will reduce health disparities.

### Objective of the study

We aimed to design and evaluate a novel training program using VR to augment and assess its impact on empathy among White-identifying HCPs toward patients identifying as BIPOC. The results of this study informed preliminary assessments of the effectiveness, acceptability, and feasibility of the intervention in real-world settings described here.

## Methods

In this pilot study, we developed VR scenarios allowing HCPs to experience the perspective of patients from a different race/ethnicity to understand better how those patients experience healthcare engagement. The VR scenarios developed for the intervention, their co-creation with input from the study’s community advisors, and the full qualitative evaluation of their impact are described previously [14]. In the first VR scenario, study participants enter the virtual clinic as a Black woman experiencing microaggressions and dismissive responses (e.g., regarding the believability of her presenting with pain) from HCPs, despite presenting with concerning health issues. In the second scenario, they enter the virtual healthcare setting as a person from the Latinx community experiencing language barriers to understanding the HCP, feeling afraid, confused, and overwhelmed by questions relating to their scheduled appointment. For example, the patient navigates the experience unable to read signage through the healthcare experience. Both VR scenarios allowed participants to embody these avatars in a life-sized virtual body, which included using a head-tracked, stereo-head-mounted display [15].

Participants underwent approximately 15 minutes of VR scenarios in a structured university-classroom setting with a VR headset and paired controllers. Each participant begins with a web-based pre-VR session, which includes a pre-test and a brief module explaining key terms relevant to the VR intervention, such as implicit bias, microaggressions, and strategies to mitigate their impact. The VR-based experience begins with a 10-minute virtual orientation and tutorial where participants learn to navigate and interact with their virtual surroundings. Thereafter each participant begins the scenario by following a series of visual prompts and interacting with different non-player characters by clicking on the control menus or responding verbally to questions or cues. Following the VR-based intervention, participants return to the web-based module to revisit concepts and complete a post-test. The complete VR process spanned approximately 35 minutes. Scripts, including dialogue between the VR avatars and the other characters, can be viewed at the URL link: https://www.youtube.com/watch?v=g4cReOX3wsI. The study was approved by the ethical review committee at UCSF, San Francisco [20–33177]. Informed consent was obtained from all participants.

### Sample

We recruited practicing clinicians and graduate learners (e.g., resident physicians and student nurse practitioners) working at university-affiliated clinical sites in San Francisco to participate in the VR training intervention from Sept 8, 2021 – February 10, 2022. Recruitment occurred through posters in communal spaces at clinical sites, as well as through Twitter posts and LinkedIn Ads. Inclusion criteria included (1) being White-identifying, (2) having a university affiliation, and (3) being a provider directly involved with patient care, which included physicians, nurses, physician assistants, and a speech-language pathologist. Exclusion criteria included (1) self-identifying HCPs not currently engaged in direct patient care, (2) HCPs self-identifying as BIPOC, and (3) anyone unable or unwilling to participate in the VR training intervention for any reason. A convenience sample of 40 healthcare professionals were enrolled in the program, and 27 completed both VR modules and completed the pre- and post-training assessment. All participants gave written consent; all data collected was subsequently de-identified although the study team assigned pseudonyms for each participant when reviewing transcripts of their interviews.

### Measures

All participants completed an initial survey; participants’ general demographic information, including age, gender, professional cadre, race, and prior diversity, equity, and inclusion training experience (DEI), was recorded. In addition, all participants were asked to complete pre- and post-participation empathy assessments, which included a situational empathy scale and the Jefferson Empathy Scale (JSE) [16]. The situational empathy scale, developed for this study, assessed providers’ empathic response to patients from historically marginalized groups. This instrument was adapted from the Rape-Victim Empathy Scale [17] and the Add-Response Empathy Scale [18]. The instrument assesses healthcare providers’ empathic response to BIPOC patients and consists of three domains that measure Perspective Taking, Compassion for the Patient, and Walking in the Patient’s Shoes.

The post-participation assessment occurred within 24 hours of the VR experience. A subset of participants also completed individual semi-structured interviews two weeks after exposure to the VR scenarios. These one-hour long, remote interviews were audio recorded, transcribed in Temi, and analyzed in Dedoose, a qualitative analytic program, utilizing thematic analysis [19]. An all-female BIPOC study team discussed the exact definition of each code and incorporated an example of a quote from transcripts into the codebook. Using iterative group discussions, the study team developed a codebook using a priori codes and incorporated in vivo codes after reviewing a sample of transcripts together. The codebook was finalized and purposefully limited to 50 codes for ease of analysis. The team then coded a subset of interviews for inter-rater reliability, before individually coding transcripts. Researchers generated themes using team-based analysis and discussions of memos. This manuscript represents findings from a subset of previously coded data. Descriptive statistics were utilized to summarize the data, and independent t-tests were conducted to compare the characteristics of participants who completed the virtual reality (VR) experience with those who did not.

### Analytic strategy

The RE-AIM framework [20] was used to evaluate the Reach, Effectiveness, Adoption, Implementation, and Maintenance of this pilot program. Referring to original definitions, *Reach* was interpreted as the absolute number and basic demographic information of those attracted by the program publicity activity and who participated in all intervention elements. The *Effectiveness* was measured by the changes in empathy scores before and after participation among those who participated in the intervention. The *Adoption* was evaluated by the perspectives of relevant participants and personnel gathered through semi-structured qualitative interviews. We interpreted the *Implementation* by the degree of intervention completion. *Maintenance* was assessed through qualitative responses, focusing on the retention of ‘empathic’ clinical practice among participants following their training. Long-term follow-up with participants was not conducted.

## Results

### RE-AIM evaluation

#### Reach.

**Fig 1:**
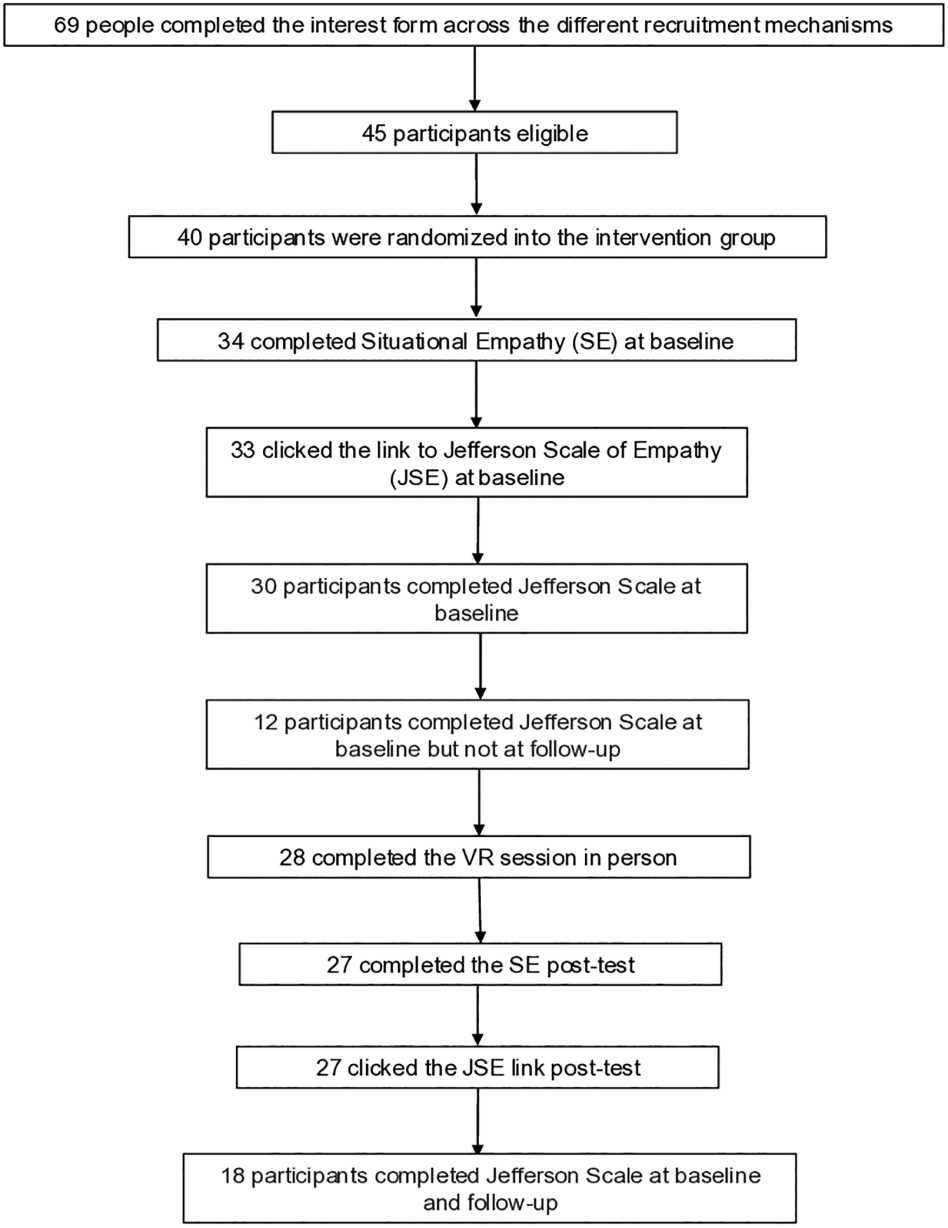
Total number of potentially interested participants who enrolled and completed each step of the VR assessment process.

The program recruited 45 eligible healthcare professionals; of these, 30 completed the JSE at baseline Fig 1. Total number of potentially interested participants who enrolled and completed each step of the VR assessment process, of whom 47% (N = 14) were physicians, most were aged between 35 and 44 years (53%, n = 16) and most were female (70%, n = 21). Notably, only 18 of those participants completed the entirety of the VR interventions and both pre- and post-assessments. Among those that completed the VR intervention and both pre-and post-intervention assessments, 72% (n = 13) were female, and 50% (n = 9) were physicians. As shown in Table 1, there was no statistical difference in age, gender, or professional cadre between those that completed the intervention and those that enrolled but did not complete it.

**Table 1:** Comparison of participants who enrolled and those that completed the study intervention and pre- and post-VR empathy assessments.

	Total	Enrolled but not completed VR	Enrolled and completed VR	p-value
	n = 30	n = 12	n = 18	
**Gender – Female**	70% (21)	67% (8)	72% (13)	0.74
**Age group**				0.86
25-34y	27% (8)	25% (3)	28% (5)	
35-44y	53% (16)	50% (6)	56% (10)	
45-74y	20% (6)	25% (3)	17% (3)	
**Race**				
White	100% (30)	100% (12)	100% (18)	
**Role at UCSF**				0.24
Physician	47% (14)	42% (5)	50% (9)	
Nurse Practitioner	7% (2)	17% (2)	0% (0)	
Fellow/Resident	10% (3)	17% (2)	6% (1)	
Student Nurse	7% (2)	0% (0)	11% (2)	
Others	30% (9)	25% (3)	33% (6)	
**Years in clinical practice**				0.72
0-4y	20% (6)	25% (3)	17% (3)	
5-9y	27% (8)	33% (4)	22% (4)	
10-14y	27% (8)	17% (2)	33% (6)	
15-44	27% (8)	25% (3)	28% (5)	

Data are presented as column % (n); p-value are for testing the differences between the two groups.

#### Effectiveness.

The average JSE score of the 18 participants was 47.2 (95% Confidence Intervals [CI] 43.9, 50.9) at baseline. It decreased slightly in the follow-up assessment after the completion of the intervention, 43.6 (95% CI 39.7, 47.5), although the change was not significant (p = 0.101). There was no significant difference between pre-and post-JSE scores, when stratified by gender. The difference in pre- vs. post-JSE scores was insignificant among participants aged 25–34 [n = 8] or 45–74 years [n = 6]. However, there was a significant difference in participants aged 35–44 years [n = 16]; the mean pre-VR score was 48.4 (95% CI 42.5, 54.3) compared to the mean post-VR score of 42.2 (95% CI 38.3, 46.1) in this age group (p = 0.004).

#### Adoption.

Semi-structured interviews with 18 study participants were undertaken after the completion of the intervention, including eight physicians, two nurse practitioners, six nurses, and two student nurses. All interviews were conducted remotely using Zoom video software and lasted approximately 60 minutes. Participants expressed a high willingness to adopt the VR training program to their ongoing professional development, as illustrated:

“*I think it would be a great addition to our yearly competency*” (40-year-old, White female nurse).

“*And the VR training, you know, by design, it’s interactive, it forces you to be there, and it forces you to have some self-reflection, and it’s not just like, okay, let me just find the multiple-choice answer that doesn’t sound ridiculous, and there I’m done*.” (43-year-old White female nurse).

“*I think it makes sense that it would be a standard part of education. Um, especially at least in my program, we have very limited like simulations*” (27-year-old, White female midwifery student).

Some participants expressed a belief that the program had improved their awareness of patients’ experiences of enacted racism in clinical practice:

“*I’ve gone through all these other trainings where you… read the story…but it’s very different seeing the words on a page of like… you can get it theoretically, but you don’t get it emotionally in the same way that…I did from that VR experience*” (34-year-old White female physician).

“*I loved the VR training…it actually puts you in…in the shoes of someone who is trying to access care, which I do not think any other platform can really provide that*” (33-year-old, White female nurse).

“*It’s the closest I’ve been to like seeing anything through somebody else. I mean, you can think about things and go, oh, they must feel really sad, but like being in that is so different…I know you’re just standing in a room with the headset on, but like, doesn’t seem that way, you know, it feels more like it than it does…Seeing like that, all of that was really impactful*” (40-year-old, White female nurse).

We asked participants the question “What *kind of training or intervention do you think would help providers reduce the impact of implicit bias on patient care?”* Several participants expressed concern that the time commitment to participate in the training might preclude the participation of other healthcare professionals.

“*For me, the VR and I realize that’s expensive and time-consuming. We have so many people that need to have this training*” (67-year-old White female nurse)

“*I think there’s definitely a place for* [a VR-based DEI training for continuing education]*. I think it’s really useful. But people are already really overworked. And so, unless you offer some kind of incentive or you make it mandatory, I think you’re gonna have trouble finding people to be interested in it. <laugh> not because of the content, but just because of the time*” (31-year-old, White/Hispanic nurse)

Participants had the opportunity to indicate any perceived positive impacts through a post-VR survey using a scale from 1–10 on how likely they would recommend the intervention to a friend. 26 out of the 28 (93%) participants who participated in the VR simulation and completed the survey indicated a favorable response of 6 or higher.

#### Implementation.

The implementation of this pilot training program is shown in Fig 1. The program was completed in accordance with the study protocol. Notably, of the 45 eligible candidates, 40 participants signed up to participate, but only 30 completed the pre-intervention JSE assessment, 30 of these participants completed the VR training sessions, but only 18 completed both pre- and post-intervention JSE assessments and the VR training experience.

#### Maintenance.

After the training program was completed, there was no longitudinal impact assessment among program participants. As such, we could not assess the intervention’s impact on clinical care among healthcare providers. Nonetheless, in semi-structured interviews performed immediately after the completion of the training, several participants expressed the belief that the training intervention would have a sustained impact on their clinical practice in the long term.

[Interviewer]: *How do you think the VR experience impacted the way that you interact with your Black and Latinx patients*?

“*I have become especially conscious of how it important it is to use empathetic communication, just to mitigate inherent stress that they may bring to those encounters from just their cognitive load, which is informed by all of their past experiences and their expectations and their difficulty in day-to-day life, encountering bias*” (39-year-old, White female physician)

[Interviewer]: *How has your approach to working with your Black and Latinx changed since your participation in CULTIVATE?*

“*I think my it’s impacted my like empathy…thinking more and more about making those conversations explicit and when a, a patient encounter is challenging or a patient is labeled as challenging thinking about, well, what does that really mean? And, you know, let’s tease that apart to reflect in a different context… that’s something that I’ve seen be different*” (34-year-old, White female physician)

At the institutional level, the training program was well-received by academic partners within the university. As evidence of the potential sustainable impact of the intervention, a pilot study was launched to explore the feasibility of VR training to reduce bias towards patients among nursing and medical students and separately among obstetric care providers. However, several BIPOC students raised objections to the training, given how the VR scenarios perpetuated racist norms. The students’ concerns were in response to recruitment flyers for the study. These objections have influenced the trajectory of the ongoing pilot study and how we engage and include all levels of learners regarding the studies’ development, recruitment and delivery.

## Discussion

We introduced a VR intervention as a tool to enhance empathy among healthcare professionals toward BIPOC patients and tested it in an academic healthcare facility. The RE-AIM framework was adopted to evaluate the program both qualitatively and quantitatively. A key finding was that the training intervention was notable in reaching female, middle-aged clinicians, as evidenced by participation data. This finding is remarkable since other research examining the use of VR to minimize implicit bias [21] and foster cultural humility, has demonstrated the utility of this kind of educational technology to reach younger professionals [22,23]. We define implicit bias as the unconscious negative attitudes against a specific social group shaped by experience and based on learned associations between particular qualities and social categories, including race and/or gender [21]. Our qualitative data also highlighted how participants felt that the intervention would have a sustained impact on their clinical practice. These data support the use of VR as an adjunct to other evidence-informed training strategies to address racism in clinical settings, such as cultural competency training [24–26] and educational initiatives that increase critical reflection on attitudes, and beliefs and increase reflexivity [27–29].

Notably, this pilot program did not demonstrate a significant impact on objective measures of cognitive empathy, the ability to understand another person’s feelings without experiencing the feelings or situational empathy, an empathic reaction in a specific situation. Moreover, the drop-off in participants between enrollment and post-intervention assessment completion, suggests that this VR intervention was challenging to fully implement. We speculate that the tools for evaluation, including the JSE tool may have been a barrier to successful implementation. Importantly, changes in JSE scores did not align with qualitative feedback from participants after participation: most respondents reported that the VR enhanced their own empathy towards historically marginalized racial/ethnic groups. The discrepancy between the JSE scores and the qualitative analyses warrants further exploration, not least because it is at odds with other studies evaluating VR to reduce implicit racial bias from White-identifying individuals toward BIPOC individuals [6,22,30]. At the very least, it suggests that the JSE tool developed to assess cognitive situational empathy [16], may not be predictive of the empathy elicited by the VR training experience, a training modality evidenced to produce changes in emotional empathy.

This study had certain limitations. First, this is an exploratory pilot study and due to the absence of a control group, we could not evaluate the effect of the program in comparison to other anti-bias racism initiatives. Cultivating racial empathy among clinical providers may demand a diversity of various educational interventions. Secondly, due to the lack of a long-term follow-up survey, we have not evaluated the sustained impact of the VR program. Moreover, we could not assess the program’s effect on clinical outcomes, as measured by patient feedback or clinical metrics. Thirdly, the sample size was small in this pilot study, which may be attributed to the study being launched during the COVID-19 pandemic in which providers had significant demands on their time. The results provided only a preliminary estimate of effectiveness that should be assessed in a larger trial and thus results should be interpreted with caution. Finally, we acknowledge that failure to demonstrate a significant impact on objective measures of cognitive empathy may reflect limitations with the JSE scale used to measure changes in healthcare providers’ emotional empathic responses [31]. This was a small pilot study; a larger sample size in a randomized controlled trial is needed to establish the efficacy of this training modality as well as more extensively evaluate the JSE and other existing empathy scales to measure situational and cognitive empathy for use in this context [32].

## Conclusion

This project was intended to evaluate the feasibility of using racism-focused VR experiences to augment existing diversity and equity training initiatives and reduce racial bias among clinical healthcare providers. Our data suggests that VR is a tool worth further exploration to augment diversity training for clinicians. The ability of VR training to change clinical practice and ultimately end health disparities will require rigorous controlled trials with acute and chronic disease endpoints. We demonstrated that VR is a feasible tool that can be integrated into provider approaches to respectful patient-centered care where it may be difficult for clinicians to situate themselves in relation to their patient’s perspective. As VR becomes more ubiquitous in medical education, there are substantial opportunities to leverage the technology to mitigate the deleterious impacts of bias and advance health equity in clinical medicine.
